# COHORT PROFILE FOR THE TONGJI CARDIOVASCULAR HEALTH STUDY: A PROSPECTIVE MULTI-OMICS COHORT STUDY

**DOI:** 10.1093/geroni/igad104.3235

**Published:** 2023-12-21

**Authors:** Ting Xu, Yueqi Lu, Bangwei Chen, Chenxin Deng, Yucong Zhang, Lei Ruan, Tao Li, Cuntai Zhang

**Affiliations:** Tongji Hospital, Wuhan, Hubei, China (People’s Republic); BGI-Shenzhen, Shenzhen, Guangdong, China (People’s Republic); BGI-Shenzhen, Shenzhen, Guangdong, China (People’s Republic); Tongji Hospital, Wuhan, Hubei, China (People’s Republic); Tongji Hospital, Wuhan, Hubei, China (People’s Republic); Tongji Hospital, Wuhan, Hubei, China (People’s Republic); BGI-Shenzhen, Shenzhen, Guangdong, China (People’s Republic); Huazhong University of Science and Technology, Wuhan, Hubei, China (People’s Republic)

## Abstract

The Tongji Cardiovascular Health Study aimed to further explore the onset and progression mechanisms of cardiovascular disease (CVD) through the combination of traditional cohort study and multi-omics analysis including genomics, metabolomics and metagenomics. This study included participants aged 20-70 years old from Geriatric Health Management Center of Tongji Hospital of Tongji Medical College of Huazhong University of Science and Technology. After enrollment, each participant underwent a comprehensive series of traditional and novel cardiovascular risk factors assessments at baseline, including questionnaires, physical examinations, laboratory tests, cardiovascular health assessments, and biological samples collection for subsequent multi-omics analysis. A biennial follow-up will be performed for ten years to collect the information above and the outcome data. Totally, 2,601 participants were recruited in this study (73.4% men), with a mean age of 51.5±11.5 years. They were mostly highly educated (college and bachelor’s degree or above: 92.8%) and mostly had healthy lifestyle behaviors. A total of 50.5% and 14.9% of men and 23.1% and 3.4% of women were overweight and obese, respectively. The proportion of several comorbidities such as hypertension, diabetes mellitus, and hyperlipidemia increased with age, and the rapid increase in women around the age of 40-50. With the progress of the cohort follow-up work, it is expected to provide unique multidimensional and longitudinal data on cardiovascular health in China.

